# The influence of nano filter elements on pressure drop and pollutant elimination efficiency in town border stations

**DOI:** 10.1038/s41598-023-46129-5

**Published:** 2023-11-01

**Authors:** Saeed Zeinali Heris, Hamed Ebadiyan, Seyed Borhan Mousavi, Shamin Hosseini Nami, Mousa Mohammadpourfard

**Affiliations:** 1 Xi’an University of Science and Technology, No. 58, Middle Section of Yanta Road, Xi’an, 710054 Shaanxi China; 2 Faculty of Chemical and Petroleum Engineering, University of Tabriz, Tabriz, Iran; 3 J. Mike Walker ‘66 Mechanical Engineering Department, Texas A&M University, College Station, TX 77843 USA; 4 School of Chemical, Biological and Materials Engineering, The University of Oklahoma, Norman, OK 73019 USA; 5 Department of Energy Systems Engineering, Izmir Institute of Technology, Izmir, Turkey

**Keywords:** Environmental sciences, Engineering

## Abstract

Natural gas stands as the most ecologically sustainable fossil fuel, constituting nearly 25% of worldwide primary energy utilization and experiencing rapid expansion. This article offers an extensive comparative analysis of nano filter elements, focusing on pressure drop and pollutant removal efficiency. The primary goal was to assess the superior performance of nano filter elements and their suitability as an alternative for Town Border Station (TBS). The research encompassed a six-month examination period, involving routine pressure assessments, structural examinations, and particle characterization of the filter elements. The results revealed that nano filters showed better performance in adsorbing aluminum than conventional filters, possibly due to their cartridge composition. Nano filters contained phosphorus, sulfur, and copper, while conventional filters lacked these elements. The disparity can be attributed to the finer mesh of the nano filter, capturing smaller pollutants. Although the nano filter had minimal silicon, the conventional filter showed some, posing concerns. Despite having 19 extra pleats, the nano filter maintained gas flow pressure while capturing more particles than the conventional filter.

## Introduction

Despite the advancements in emerging energy sources, fossil fuels continue to serve as the primary global energy source^1–4^. The excessive reliance on fossil fuels in ongoing and expanding industrial processes has led to a persistent rise in CO_2_ emissions^5^ , which is widely acknowledged as the primary driver of the escalating problem of anthropogenic climate change^6–8^. Moreover, the extensive use of coal-fired resources is exacerbating air pollution^9,10^.

Natural gas (NG) is the most environmentally friendly fossil fuel, accounting for nearly a quarter of global primary energy consumption and exhibiting rapid growth. NG is primarily transported through high-pressure pipelines, representing 67.5% of the global gas trade, and requires 3–5% of the gas to be consumed at compressor stations for pressure maintenance. Pipeline pressures typically vary depending on the distance traveled, with a range of 50 to 100 bar for distances exceeding 100 km and 20 to 50 bar for distances over 20 km. However, before supplying natural gas to local systems or end users, it is necessary to decrease the pressure to lower levels at pressure reduction stations (PRSs)^11–13^. In natural gas pipelines, the presence of particles and contaminants, such as rust, deposits, condensates, and solid particles, necessitates their removal to protect station equipment and meet gas quality standards. Long-distance transmission can introduce additional hazards like black powder and droplets, leading to corrosion, clogging, and valve deterioration.

Fouling of compressor systems can also occur, risking gas leakage and shutdown; therefore, effective filtration is essential to maintain the integrity, efficiency, and safety of the gas transmission network, preventing equipment damage and ensuring compliant delivery to consumers^14–16^. Moreover, a notable escalation in pressure drop is observed, coupled with an augmentation in dust cake accumulation. Sustaining the gas flow at the prescribed nominal flow rate through the filter enclosure necessitates providing supplementary energy. Consequently, this heightened energy requirement increases operating costs associated with the gas cleaning system. In addition, bag filters employed in high-temperature environments are susceptible to potential damage or obstruction caused by porous particles^17^.

The filter element is commonly used in dry gas filtration, typically composed of durable materials like pleated paper, synthetic fibers, or porous metal. It is designed with a large surface area and precise pore size to efficiently capture particles of different sizes. This type of dry gas filter offers several benefits, including effective particle removal, enhanced gas quality, prolonged equipment lifespan, improved system performance, and cost savings. Utilizing an efficient filter element is crucial for maintaining the reliability and integrity of natural gas systems^18^.

Over time, various types of filters have been utilized for natural gas filtration. However, nanotechnology is an emerging technology with the potential to address practical challenges faced in the oil and gas industry^19–21^. Nano filters have been highlighted in various sources for their multitude of advantages. These include operating at low pressure while maintaining high flux and effectively retaining multi-capacity anionic salts. Nano filters not only offer the advantage of relatively lower operational and maintenance costs but also possess selectivity capabilities, enabling precise filtration. Also, they are known for their reduced weight, making them more convenient to handle. Despite having small-sized pores in the filter elements, nano filters still achieve a lower pressure drop, allowing for operations at reduced operational pressures and consequently requiring less energy^22^.

Over the course of numerous years, researchers have dedicated significant efforts to enhance the performance of filters through the systematic investigation of influential parameters and cleaning intervals and the exploration of various cleaning techniques. Li et al.^23^ conducted a comprehensive experimental investigation to assess the influence of particle size and maximum pressure drop on the performance of a double-pleated cartridge filter. Through advancements in filtration technology and the implementation of pulse-jet cleaning, it was observed that the filtration cycle decreased while the residual pressure drop increased across all tested conditions. When subjected to the same maximum pressure drop, larger particle sizes exhibited reduced average pressure drop, decreased frequency of pulse-jet cleaning, and lower average dust emission concentration. Gul et al.^24^ employed grey Taguchi and artificial neural network (ANN) optimization methods to enhance gas turbine performance. Through ANOVA analysis, they found that air-inlet temperature had the most significant impact (71.17%) on the output parameters, while the type of air-inlet filter had the least effect (1.40%). This efficient optimization methodology can be applied to optimize process parameters in gas turbines within the power generation industry, leading to improved performance and efficiency. In a separate study conducted by Li et al.^25^, the impact of nozzle optimization beneath an injection pipe in a pulse-jet cartridge filter was examined. The results revealed that, following the optimization process, there was an increase in peak dust emission concentrations; however, the average dust emission concentration decreased for the same filtration interval or duration. They further asserted that both the average residual filtration pressure drop and average filtration pressure drop exhibited a decrease, while the filtration interval experienced an increase. Kim et al.^26^ conducted a study that measured the effective filtration area of a pleated bag filter designed explicitly for pulse-jet cleaning. Their investigation revealed a strong dependence of the filtration area and filter cleaning efficiency on the filter’s pleating geometries. A dimensionless parameter, α, representing the ratio of pleat height to pleat pitch, was introduced to determine the optimal pleat geometry. It was observed that higher α values resulted in larger filtration areas, thereby enhancing the overall performance of the filter. In a study by Su et al.^27^, a hierarchically structured nanofibrous material was successfully synthesized and investigated for its efficiency in removing toluene and particulate matter (PM). The researchers determined that these nanofibers exhibited superior effectiveness compared to competing materials, substantiating their potential application for air filtration purposes. Li et al.^28^ explored the utilization of a waterproof-breathable PTFE nano- and microfiber membrane as a high-efficiency filter. Experimental outcomes presented notable enhancements in filtration efficiency when targeting PM2.5 particles, increasing from 44.778 to 98.905%. On the other hand, the filtration efficiency for PM7.25 particles reached 100% following the integration of the electrospun PTFE nanofiber layer. These findings highlight the potential of incorporating nanoparticles in filter materials to effectively address indoor and outdoor dust removal challenges and cater to gas filtration requirements in industrial settings.

Based on the extensive literature review conducted, it is evident that the focus has primarily been on improving filtration efficiency through cleaning techniques and optimizing operational conditions. However, to the best of our knowledge, none of the studies explored the implementation of nanotechnology to enhance filter performance. Drawing upon various relevant sources, this project marks the first simultaneous comparison of two filter elements with differing structural characteristics at an urban pressure reduction station called TBS (Town Border Station).

Hence, this scientific and practical study aims to compare the existing filter elements utilized by the East Azerbaijan Gas Company with a newly proposed nano filter element. With the numerous benefits associated with nanotechnology, the company seeks to assess the advantages of these nano filter elements in operational conditions, compared to their previous filter elements, and determine whether a replacement is warranted. The evaluation encompasses weight analysis, surface contact area, SEM, and other analyses to assess the efficiency and performance of the two filter elements in relation to each other.

## Materials and methods

### Experimental system and associated methodologies

**Figure 1:**
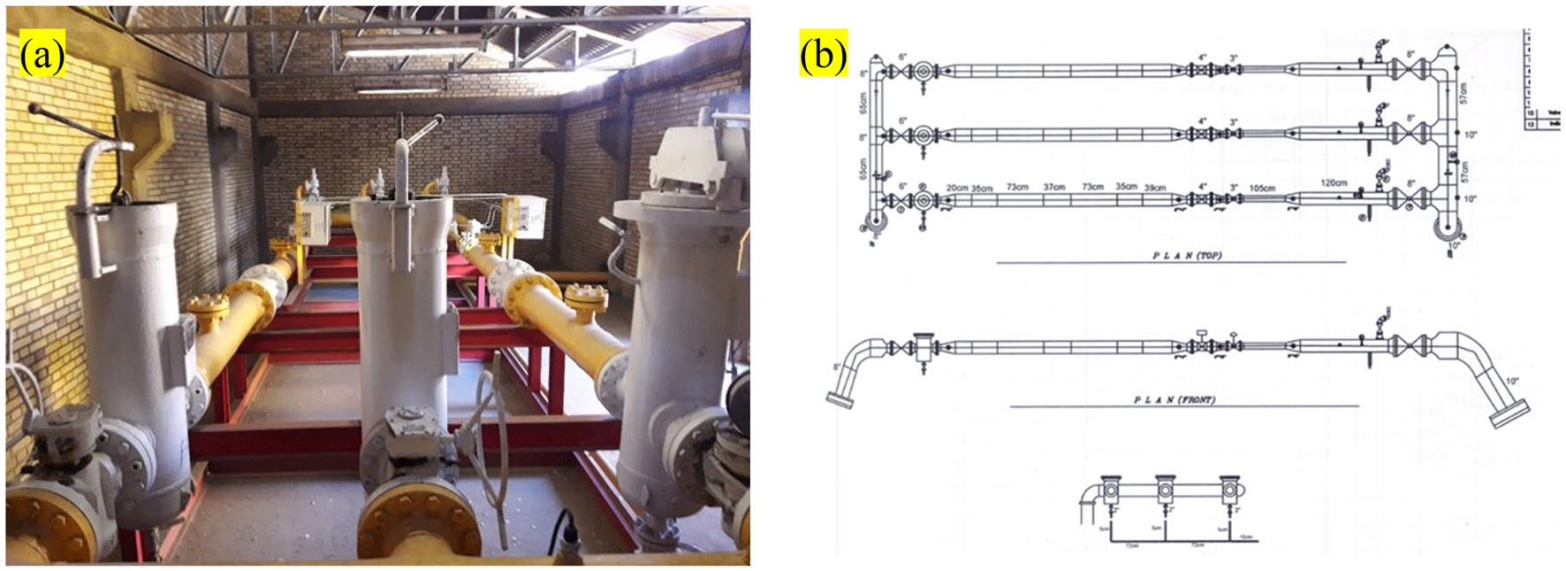
(**a**) TBS station, and (**b**) layout map of station.

This study aims to investigate and compare the performance of a newly proposed nano filter element with the previous filter elements employed by the gas company. A TBS is required within the East Azerbaijan province to facilitate the necessary experiments. Through comprehensive research and analysis of the technical specifications of various stations across the province, TBS station number 522 in Tabriz City was specifically chosen. This station has a maximum capacity of 20,000 cubic meters, as illustrated in Fig. 1a. A general layout map of the installed infrastructure at the aforementioned station is provided in Fig. 1b. The station consists of three units: an active unit, a monitor unit, and a bypass unit. In order to establish uniform operating conditions for the parallel testing of the filters, they were selected based on matching characteristics and operating conditions. Furthermore, the gas inlet flow rate and pressure were set consistently within the maximum tolerance range. This approach was necessary due to variations in gas characteristics throughout the different seasons. It is worth noting that gas consumption differs across seasons. To assess the physical durability of the nanotechnology-based filter element and ensure its optimal performance under peak operational conditions, the winter season was chosen as the testing period. Considering the increased gas consumption during winter, a higher volume of gas passes through the filters. The gas company’s instructions recommend replacing the filter elements every 6 months. Hence, the testing period was 6 months to align with the replacement schedule. However, to minimize potential errors, it was decided to conduct evaluations and interchange the filters every 3 months. Another crucial parameter in evaluating the filter elements is the pressure drop, which refers to the pressure difference between the gas inlet and outlet. As previously mentioned, the gas flow enters the filter container through small holes in the filter element. After the filtered gas adsorbs pollutants, it proceeds to the gas transmission lines. Therefore, the filter element should be designed to impose minimal pressure drop on the system.

Simultaneously with the practical testing, a corresponding sample of the new nano filter element was sent to the accredited laboratory at Isfahan Technology City. This laboratory is recognized as a reliable center by the gas company, and its findings are included in the results section of this study. The pressure difference is a vital indicator for the timely replacement of filter elements. Occurrences such as heightened pollutant adsorption or the ingress of atypical physical objects into the gas transmission line can obstruct gas flow through specific regions of the filter element, causing blockages and subsequent pressure drops. In certain cases, this phenomenon may create an alternate pathway for the gas flow, bypassing the intended filtration process within the filter element. Differential pressure gauges are employed to measure the pressure differential between the gas inlet and the filter outlet. These gauges, featuring red and black hands, are permanently affixed to the filters to provide continuous monitoring and accurate assessment of the pressure differential, facilitating appropriate maintenance and replacement decisions. After approximately six months, the filters were carefully removed in collaboration with technicians assigned by the East Azerbaijan Gas Company. Following safety guidelines, standard practices, and company instructions, the gas was shut off before extraction. The filters were then transferred to pre-prepared chambers to minimize contact with pollutants and maintain a controlled environment. Upon arrival at the laboratory, a series of tests were initiated to compare the performance of the two filters. The first method involved assessing the pollutants adsorbed by the filters through pre- and post-weighing the filters after their usage in the pressure reduction station within the city. The second method entailed conducting objective visual inspections of the filters following their deployment in the stations.

### Specifications of cartridge filter elements (metal) supplied by the gas company to filter element manufacturers

The media of these filters typically consist of non-woven industrial felt made from polypropylene or polyester fibers. These fibers are needle-punched and heat-set, forming a structured pattern and reinforced with galvanized wire mesh. The media must possess hydrophobic properties, as this factor is essential for their intended application in the filter. This factor is significant because if the hydrophobic property is not considered in the media, the filter element will be incapable of capturing liquids in the gas stream. Therefore, experiments were conducted on filter elements produced using nanotechnology to confirm the presence of this property in the mentioned filter. To demonstrate this, one of our experiments involved placing a dry adsorbent paper inside the inner diameter of the filter element and securing it in place. Subsequently, 100 mL of water was carefully poured onto the outer diameter of the filter, and ample time was provided for the water to thoroughly saturate the filter. Fortunately, no traces of the liquid were observed inside the filter element.

The body of the filter was affixed to the mesh using the linear heat bonding technique, eliminating the need for stitching or glue (surface heat welding method). The adhesive used to connect the rings and the filter element was positioned within the thermos bracket, ensuring a secure and leak-proof connection. The adhesive had a thickness of 1 mm. The thickness of the inner and outer body sheets for class 150 and 300 filters was determined based on the filter height. For filters with a maximum height of 45 cm, the outer body had a thickness of 0.6 mm, while the inner body had a thickness of 0.7 mm. However, for filters taller than 45 cm, the outer body remained at 0.6 mm, while the inner body was curved along the length of the inner cylinder and had a thickness ranging from 0.8 to 0.9 mm. For class 600 filters with a height exceeding 45 cm, the cartridge’s inner and outer body sheets had specific thicknesses. The outer body was 0.8 mm thick, while the inner body was 0.9 mm thick. The inner body was curved along the length of the inner cylinder to achieve the desired shape. For dimensions less than 45 cm, it was necessary to consider a thickness of 0.6 mm for the outer body and 0.8 mm for the inner body.

The gasket material was manufactured using NBR (nitrile butadiene rubber), with a minimum thickness of 5 mm and a shore-A hardness of 60–65. It is worth mentioning that gaskets made of sponge or carpet tires were not approved. The internal and external sheets, rim, and cap were constructed using a carbon steel sheet that successfully passed the 12 to 15 h steam bath test, ensuring a thickness of 5 μm. The micron rating of this particular type of cartridge filter ranged from 5 to 7 μm. For cartridge filters, it is essential to employ surface (stacked) filtration to achieve maximum media efficiency, longer service life, and high filtration performance. The use of depth filtration by the twisting method is not approved for these filters, as it limits the filtration surface to only the first and second layers of the filter winding surface. Both element filters adhere to the aforementioned specifications and IGS (Iranian gas standards), and the utilized nano filter element was provided by Azad Filter Company.

**Figure 2:**
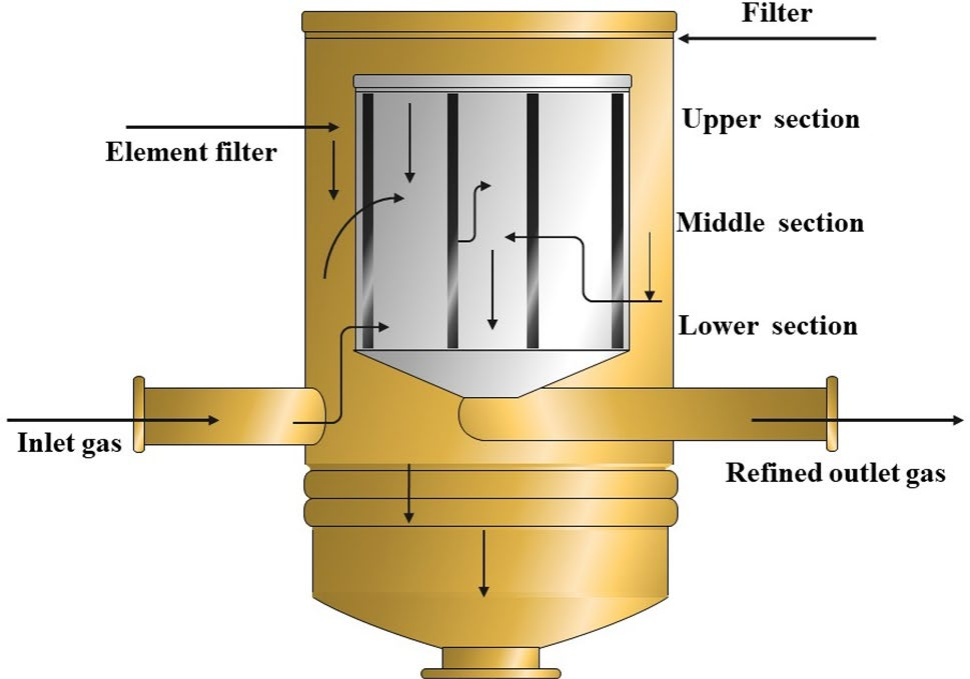
The dry gas filter and its internal element.

The two filter elements were analyzed and compared based on mesh size, outer and inner diameter, length, number of folds or pleats, and other relevant parameters (Table 1). Subsequently, both filters were divided into three sections and each of these sections was cut accordingly. These sections are referred to as the lower, middle, and upper sections, as depicted schematically in Fig. 2.

**Table 1:** The technical details of the nano filter elements.

Technical details	
Filter’s type	Filter elements fabricated using nanotechnology
Dimensions (mm)	Length: 570, outer diameter: 200, inner diameter: 130
The internal protective layer	Galvanized carbon steel
The external protective layer	Galvanized carbon steel
Thermal resistance	70 °C
Efficiency	99.9%

The lower part serves as the entry point for the gas into the filter compartment. Within this region, the gas undergoes filtration as it passes through the filter element, and the filtered gas is subsequently discharged. The samples obtained from this area were subject to analysis utilizing SEM and high-energy X-ray spectrometry. The results present the results of the pollutants adsorbed by the filter layer and the corresponding elemental composition percentages.

### The operational lifespan

According to the gas company’s standards, the filter elements should demonstrate a minimum recorded 6 months, indicating maximum operational efficiency and longevity in the flow line. Due to their undamaged structure and absence of excessive contamination, both filter element samples in this section have proven to have an excellent operational lifespan.

### The prime cost

The product’s price was another crucial and influential factor to consider. It was essential to ensure that the price difference was reasonable and justified, considering all the product’s advantageous features for the respective gas company. Even though the new nano filter element could adsorb more and had a longer lifespan, the price difference needed to be justifiable. This would determine whether the Gas Company of East Azerbaijan Province would be willing to replace the old filter element with the new nano filter element.

### The minimum size of particles trapped in the filter element

The notion that a filter element’s effectiveness for a gas company increases with its ability to capture smaller particles is flawed. Existing standards indicate that if the pores of a filter element are smaller than a certain threshold, it results in significant pressure build-up along the fluid path. Furthermore, as anticipated, the filter element is prone to tearing and disintegration under the pressure of alternating gas flow. Research findings revealed that the minimum size of particles trapped by the filter element should not exceed 3 μm to prevent clogging. Ideally, a 3–5 μm range was deemed appropriate, whereas particles larger than this threshold resulted in elevated equipment corrosion.

### The number of pleats

This factor contributed to an augmentation of the filter’s surface area, thereby enhancing the filtration level within the confined space of the filter element. However, if multiple levels of pleats were present, they could result in a pressure drop along the flow path. Thus, it was imperative to exercise caution to avoid excess pleats. An investigation was carried out to analyze the number of pleats in the two examined filters, and this data is presented as a comparative factor in the results.

## Results and discussion

### Compliance testing with standard

This section incorporates the findings of the Isfahan Technology City Laboratory, along with associated documents and relevant analyses, such as SEM and XRD, to identify pollutants associated with nano and conventional filter elements. Three distinct filter sections were dissected and examined to achieve this objective: the gas inlet section of the filter element, the middle section of the filter element, and the gas outlet section. In compliance with the IGS-M-PM-111(1) standard, the filter element crafted utilizing nanotechnology underwent a comprehensive examination, presented in Table 2. The subsequent findings revealed that the filter element had been manufactured in accordance with the standard and had received approval based on these criteria. Moreover, the performance evaluation of the nanotechnology-based filter element demonstrated a notable advantage in terms of average efficiency when compared to conventional filters. The technical results provided by the manufacturer, Azad Filter Company, exhibited a satisfactory concurrence with the test outcomes.

**Table 2:** The test results aligned with the IGS-M-PM-111(1) standard.

Clause	Test	Result	Acceptable range	
	Initial pressure drop (pa)	150	≤ 250 pa	
	Initial efficiency (%) at (4.5 to 5.5)	86.3%	≥ 60%	
	Average capacity (g) at 650 pa	99.9%	≥ 95%	
	Collapse test (psi)	45	45	
Tested device				
Model	Manufacturer	Construction	Filtration area	Filter dimensions
Dry gas filter	Azad filter	Cylindrical element	1.03 m^3^	OD:200, ID:130, H:474 mm
Test data				
Air flow rate	Air temperature	Air RH	Aerosol	Loading dust

Table 2, the subsequent findings revealed that the filter element had been manufactured in accordance with the standard and had received approval based on these criteria. Moreover, the performance evaluation of the nanotechnology-based filter element demonstrated a notable advantage in terms of average efficiency when compared to conventional filters. The technical results provided by the manufacturer, Azad Filter Company, exhibited a satisfactory concurrence with the test outcomes.

### Conducted weighing and visual analyses

**Figure 3:**
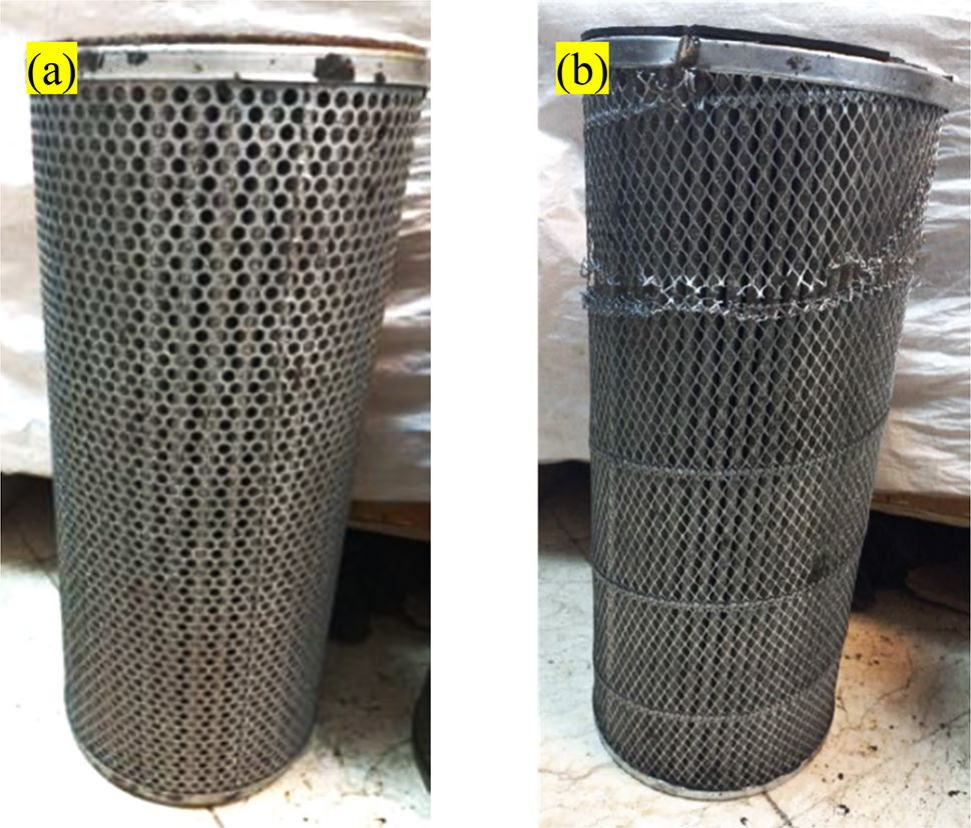
(**a**) Nano filter element, and (**b**) conventional filter element after six months of utilization.

Figure 3 pertains to the nano filter after 6 months of utilization in the TBS, and the conventional filter after six months of usage in the TBS. Upon sectioning, a visual examination revealed that the nano filter exhibited higher pollutant adsorption than the conventional filter. An additional approach employed to validate the current study’s findings and determine the quantity of adsorbed pollutants involved weighing the filters before and after their utilization at the station. Based on the obtained results, it was observed that the nano-element filter weighed 75 g, while the typical filter element weighed 33 g; consequently, the nano filter element eliminated 42 g more compared to the typical filter element over six months. Table 3 presents the results of the weight monitoring for the filter element.

**Table 3:** The results of weight monitoring of filter elements.

Filter type	Initial weight (kg)	Weight after filtration (kg)	Amount of adsorbed pollutant (g)
Conventional filter element	3.700	3.733	33
Nano filter element	4.525	4.600	75

### Pleats and pressure drop

As elucidated in the preceding chapter, the number of pleats in a filter element directly impacts the filtration level, with a higher pleat count leading to an increased adsorption rate. However, it is essential to recognize that there is a limit to the number of pleats, as consideration must be given to potential issues such as pressure drop and filter element blockage. The conventional filter element had 36 pleats, while the nano filter element comprised 55 pleats. The nano filter element exhibited 19 more pleats than the conventional filter element. To assess the pressure drop, periodic measurements of the disparity between the inlet and outlet pressures of the station were conducted and recorded at the commencement and conclusion of each run^29,30^.

To assess the pressure drop, periodic measurements of the disparity between the inlet and outlet pressures of the station were conducted and recorded at the commencement and end of each run^31^ . Based on the acquired findings (tabulated in Table 4), it can be deduced that the nano filter did not induce an excessive pressure drop, despite extra pleats and nanostructure, which stands as both advantageous and commendable^32,33^.

**Table 4:** The pressure differential recorded between the two ends of the elements.

Time of testing (month)	Pressure drop for conventional filter (PSI)	Pressure drop for nano filter (PSI)
0	5	5
1	6	6
2	8	8
3	10	10
4	10	10
5	10	10
6	10	10

### Characterization

**Figure 4:**
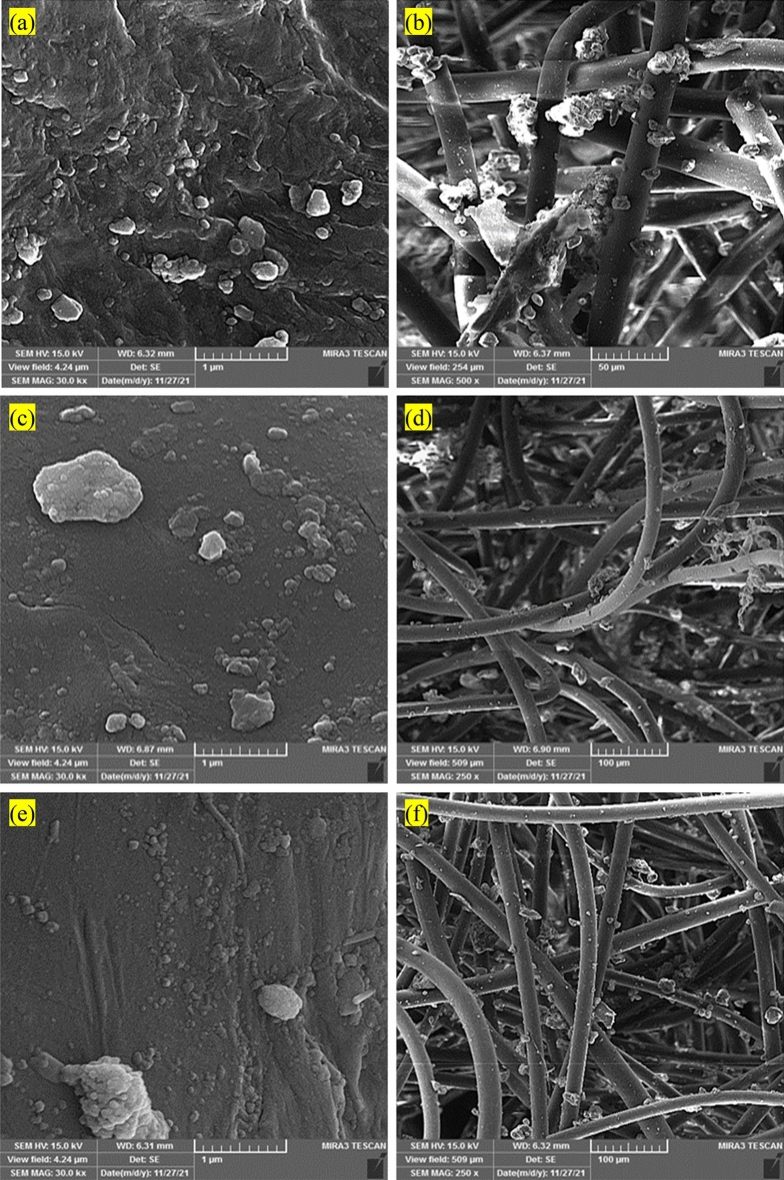
SEM images of the (**a**,**b**) lower section of the nano filter, (**c**,**d**) middle section of the nano filter, (**e**,**f**) upper section of the nano filter.

SEM analyses were performed to examine the structural and morphological characteristics of the particles within the nano filter^34–38^. Figure 4a and b depict the SEM analyses of the lower section of the nano filter, where the gas is discharged. Based on the observations made in this figure, it was noted that the surface of the nano filter exhibited a higher accumulation of pollutants compared to typical filters. The enhanced ability of nano filters to adsorb impurities in city gas will be further discussed. Figure 4c and d display the SEM images depicting the middle section of the nano filter. Through these images, the shape of the pollutants adhering to the surface of the nano filter was observed. In comparison to the lower part of the nano filter, it was noted that the middle section exhibited a relatively lower degree of pollutant adsorption. Figure 4e and f showcase the results of the SEM analyses performed on the gas outlet section of the nano filter and the pollutants found on the surface of the nano filter.

**Figure 5:**
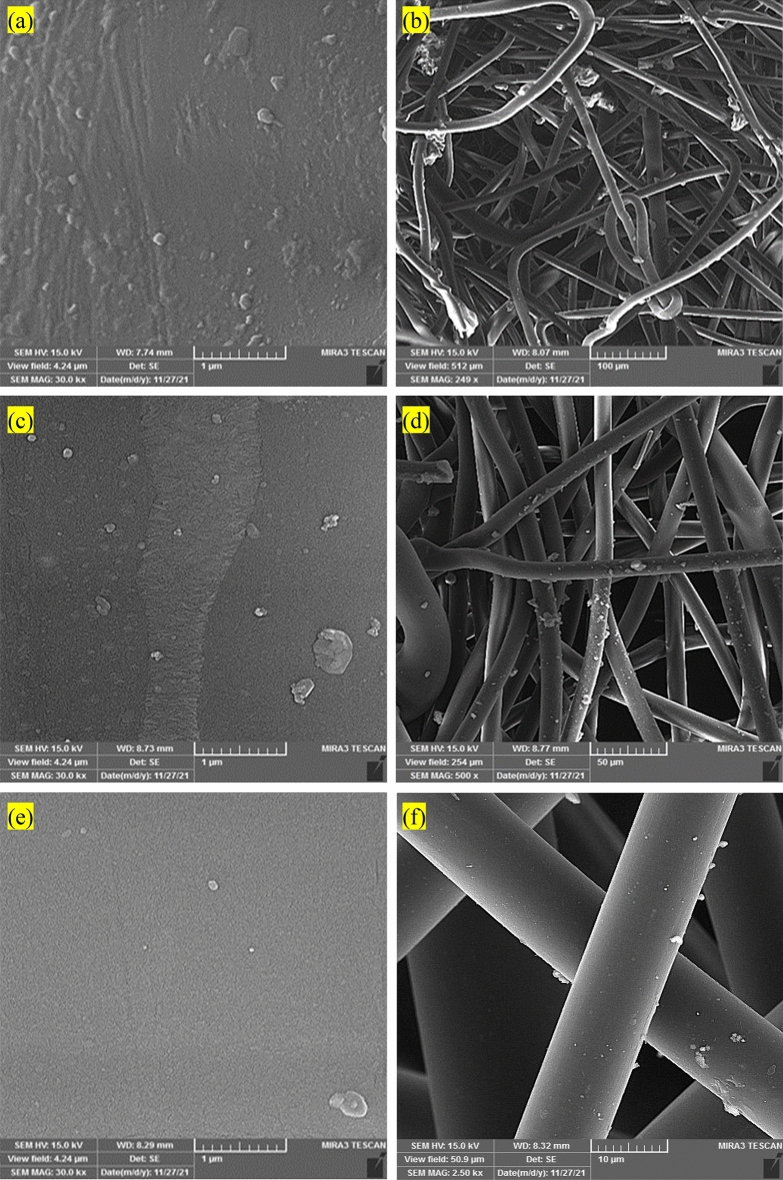
SEM images of the (**a**,**b**) lower section of the conventional filter, (**c**,**d**) middle section of the conventional filter, (**e**,**f**) upper section of the conventional filter.

In contrast to the middle section, a comparable level of pollutant adsorption was noticed in this particular region. This observation can be attributed to the gas entering the filter chamber from the bottom, where the subsequent high suction and pressure facilitate the majority of the gas exiting through the lower portion. As a result, the lower section exhibited the highest concentration of adsorbed pollutants. Figure 5a and b presents the SEM results for the gas outlet section of the conventional filter. Based on this figure, it was observed that the conventional filters exhibited significantly inferior performance compared to the nano filters over the same duration of usage. The findings obtained from the SEM analyses performed on the middle section of the conventional filter (Fig. 5c and d) further substantiated the results obtained from the same usage period. The SEM analyses on the upper part of the conventional filter, presented in Fig. 5e and f, revealed a considerably inferior performance compared to the nano filters. Notably, among the various sections of the conventional filter, the lower part comprising the gas inlet and outlet exhibited the highest degree of pollutant adsorption.

**Figure 6:**
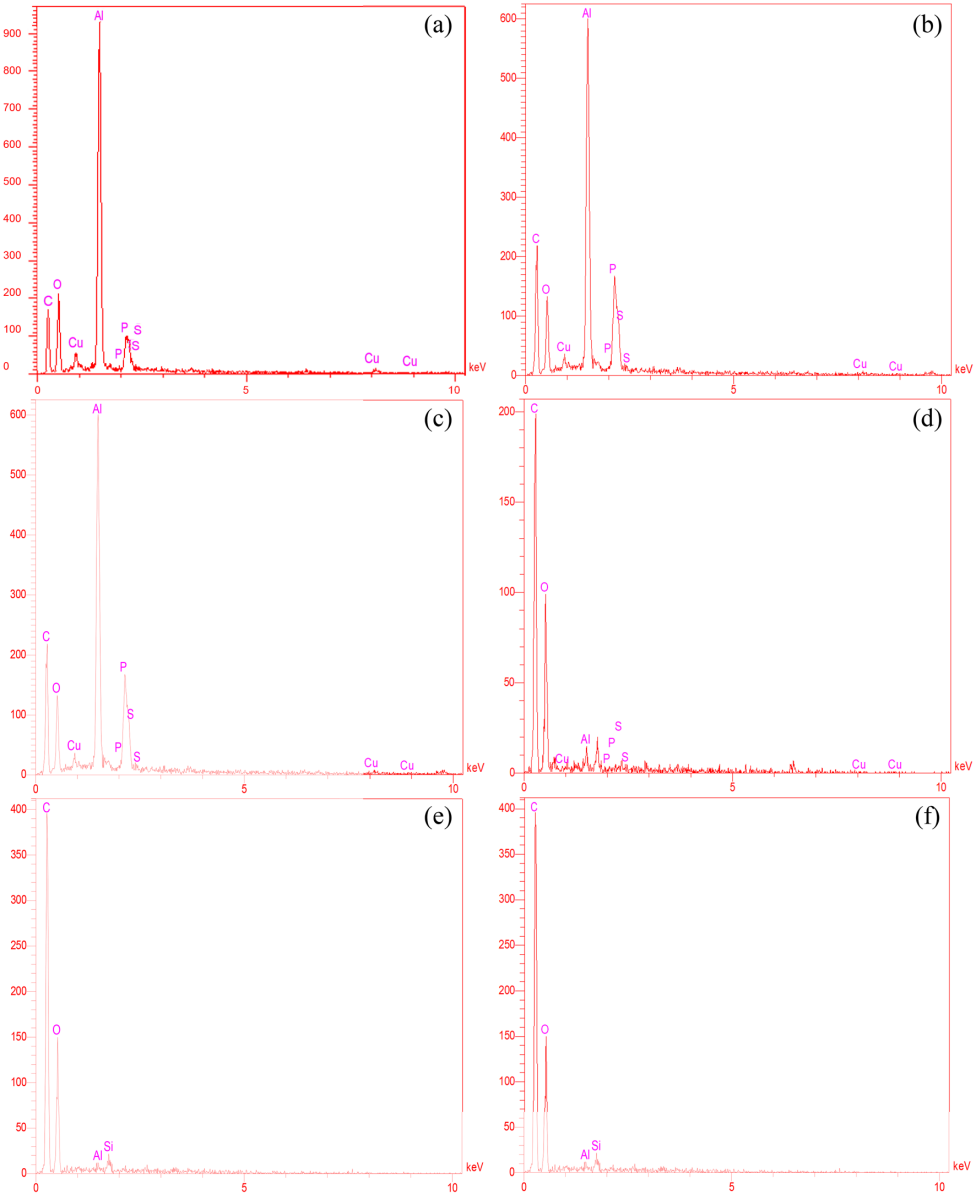
XRD results for provided samples from (**a**) lower, (**b**) middle, (**c**) upper section of nano filter, (**d**) lower, (**e**) middle, (**f**) upper section of conventional filter.

XRD analyses were performed to investigate the compositions of phases^39–42^. Figure 6a–c depicted the XRD analyses conducted on samples cut from the lower, middle, and upper sections of the nano filter, respectively. The investigation revealed the elemental composition of the pollutants within the nano filter, expressed as percentages. Based on the acquired results, the pollutants present in the lower section of the nano filter comprised the following percentages of elements: 43.39% carbon, 27.68% oxygen, 23.75% aluminum, 0.32% phosphorus, 2.77% sulfur, and 2.09% copper. The XRD analyses performed on a sample taken from the middle section of the nano filter unveiled the occurrence of various elements as pollutants. The results indicated that the pollutants comprised 54.75% carbon, 23.77% oxygen, 19.27% aluminum, 0.08% phosphorus, 0.77% sulfur, and 1.36% copper. The employed XRD analysis for the upper section of the nano filter indicated that the composition of pollutants was carbon 58.86%, oxygen 37.34%, aluminum 0.27%, phosphorus 0.18%, sulfur 0.22%, and copper 3.14%. The same characterization technique was exploited for the conventional filter’s lower, middle, and upper sections, which are illustrated in Fig. 6d–f, consecutively. According to the results obtained for the lower section of the conventional filter, the percentages of the elements in the pollutants included carbon (63.67%), oxygen (35.53%), aluminum (0.24%), and silicon (0.56%), respectively. In the middle section sample, the pollutants contained the following element percentages: 17.63% carbon, 36.47% oxygen, 0.12% aluminum, and 0.24% silicon. Based on the XRD analysis conducted on a sample taken from the upper section of the conventional filter, which determined the elemental composition percentages of the pollutants, the following results were obtained: carbon 30.62%, oxygen 37.34%, aluminum 0.13%, and silicon 0.17%. Table 5 presents a concise summary of all the quantitative XRD results depicted in the manuscript.

**Table 5:** Quantitative XRD results for nano and conventional filter’s lower, middle, and upper sections.

Type of filter	Element %						
	Carbon	Oxygen	Aluminum	Phosphorus	Sulfur	Copper	Silicon
Lower section of nano filter	43.39	27.68	23.75	0.32	2.77	2.09	–
Middle section of nano filler	54.75	23.77	19.27	0.08	0.77	1.36	–
Upper section of nano filter	58.86	37.34	0.27	0.18	0.22	3.14	–
Lower section of conventional filter	63.67	35.53	0.24	–	–	–	0.56
Middle section of conventional filter	17.63	36.47	0.12	–	–	–	0.24
Upper section of conventional filter	30.62	37.34	0.13	–	–	–	0.17

The results obtained from the study revealed that, over six months, the nano filter exhibited higher pollutant adsorption than the conventional filter. Furthermore, the lower section demonstrated a greater affinity for attracting pollutants within the nano filter than the middle and upper sections. Based on these findings, replacing conventional filters with nano filters is strongly recommended as they yield a cleaner gas output. On top of that, the nano filters exhibited a higher aluminum adsorption than conventional filters. Additionally, the nano filters displayed the presence of phosphorus, sulfur, and copper elements, which were not observed in the conventional filters. Conversely, the conventional filter elements contained a higher quantity of carbon in various sections, while silicon was detected in significantly lower amounts compared to the nano filter elements.

### Environmental aspect

Phosphorus, copper, and sulfur can be present in natural gas as impurities, typically in trace amounts. These impurities can originate from the natural gas reservoir or be introduced during extraction and processing. Phosphorus compounds are commonly present in natural gas due to phosphates found in the geological formations from which the gas is extracted. However, the presence of phosphorus in the gas stream has several disadvantages. One significant drawback is corrosion. When phosphorus reacts with moisture in the gas, it forms phosphoric acid, which can potentially corrode the metal surfaces of pipelines and equipment. This corrosion can lead to structural damage, resulting in leaks, posing a safety hazard, and requiring costly repairs or replacements. Another consequence is fouling. Phosphorus compounds tend to deposit on the inner surfaces of pipes and equipment, forming a layer of scale or sludge. This accumulation can hinder the efficient flow of gas, increasing pressure drop and impeding the smooth operation of valves and meters. The presence of fouling substances can lead to decreased system performance and necessitate frequent maintenance to remove the deposits and restore optimal functioning. Copper is introduced into the natural gas stream through various sources, including copper pipelines, compression stations, or contact with materials containing copper. However, including copper in the gas stream brings several disadvantages.

Corrosion presents a significant fault when it comes to copper. When exposed to moisture and certain impurities, copper can undergo corrosion, releasing copper ions^16^. These ions can potentially react with other gas components, particularly hydrogen sulfide, resulting in the formation of corrosive compounds. The consequences of this corrosion include pipeline and equipment damage, which can cause structural deterioration, leaks, and the need for repairs or replacements. Another shortcoming arises from the catalytic activity of copper. Copper possesses catalytic properties, meaning it can accelerate specific chemical reactions. Although this property can be advantageous in specific applications, it becomes problematic when copper is present in a gas stream. If the gas contains reactive compounds, the presence of copper can act as a catalyst, promoting the formation of undesired byproducts or triggering unfavorable reactions.

Consequently, this can adversely affect the gas quality and pose operational challenges. Sulfur compounds, including hydrogen sulfide (H_2_S), can naturally occur in natural gas reservoirs or be intentionally added during extraction. However, the presence of sulfur in the gas stream carries several disadvantages. One significant impairment is corrosion. Hydrogen sulfide is highly corrosive and can attack metal surfaces, leading to the deterioration of pipelines and equipment. The corrosive nature of sulfur compounds weakens the integrity of the gas infrastructure. It increases the risk of leaks, posing safety hazards and requiring extensive maintenance or replacement of affected components^43^.

Additionally, sulfur compounds contribute to environmental and health concerns. When released into the atmosphere during combustion or leaks, sulfur compounds contribute to air pollution. They can react with other compounds to form sulfur dioxide (SO_2_), a major contributor to acid rain and respiratory issues. Removing sulfur from natural gas through processes like desulfurization is crucial to minimize environmental impact and comply with emission regulations. Overall, including phosphorus, sulfur, and copper in the gas stream introduces challenges related to corrosion and fouling. These issues can compromise the integrity of the infrastructure, reduce operational efficiency, and increase maintenance requirements, resulting in additional costs and potential safety risks^44–47^. The utilization of nano filter elements presents a significant advantage compared to conventional filters due to their ability to effectively remove copper, sulfur, and phosphorous from the gas stream. This phenomenon can be justified by the presence of nanomaterials^48^ , which has been demonstrated to provide justification for their effectiveness in adsorption, particularly in relation to the presence of silicon in a natural gas stream. The fundamental variables contributing to the elevated adsorption rates are nanomaterials’ substantial reaction surface areas and porosity. The disparity can also be ascribed to the more delicate mesh dimensions of the nano filter component, enabling it to effectively capture pollutants of smaller sizes that would otherwise elude the conventional filter element^49,50^.

## Conclusion and future research considerations

This study aimed to validate the manufacturer’s claim regarding the efficacy of their nanotechnology filter elements used in TBS. The manufacturer asserted that these nano filters could eliminate higher amounts of pollutants from the gas stream, resulting in a significantly improved gas quality for consumers. To investigate this claim, identical filters were installed in the gas flow line for a continuous period of six months. Throughout these six months, regular and monthly checks were conducted to measure the pressure difference between the beginning and end of the filter elements. It was observed that none of the filter elements caused any abnormal pressure drops in the system. In order to simulate actual operating conditions more accurately, two filters were replaced over three months. After the standard duration of filter element placement, the filters were dissected into three distinct sections and subjected to imaging methods, including SEM and XRD analyses. The results revealed the following key findings:

1. The nano filters exhibited higher adsorption of aluminum compared to the conventional filters, potentially attributed to the material composition of the cartridge.
2. The nano filters contained elements such as phosphorus, sulfur, and copper, which were absent in conventional filters. Conversely, the conventional filters displayed higher amounts of carbon throughout various parts. This discrepancy can be attributed to the finer mesh size of the nano filter element, which allows it to capture smaller-sized pollutants that the conventional filter element cannot.
3. The nano filter element had a negligible amount of silicon, whereas the conventional filter element showed a small presence of this element, indicating a potential concern.
4. Despite having 19 more pleats, the nano filter element did not cause a significant pressure drop in the gas flow line compared to the conventional filter element. However, it effectively retained a higher quantity of particles.

Based on the investigations conducted in this research and the obtained industrial data, potential areas for future work can encompass:

1. In experiments and field investigations involving gas transmission lines, it is essential to acknowledge that uniform gas characteristics cannot always be achieved throughout the pipeline. The quality of the gas passing through may vary at different locations along the line, specifically at the beginning, end, and middle portions. When seeking to identify the highest concentrations of pollutants, it is often observed that the endpoints (beginning and end) of the pipeline exhibit the highest pollutant levels. If feasible, testing should be performed at various segments of the line to capture a more representative picture of pollutant levels and variations across the system. This approach will enable a more accurate assessment of the filters.
2. Time plays a crucial role in gas consumption, as it varies across seasons throughout the year. Conducting studies during various seasons and within the same periods, such as 6, 12, and 18 months, is advisable to gain comprehensive insights. A more thorough understanding of the fluctuations and trends can be obtained by investigating gas consumption patterns over multiple seasons and timeframes.
3. A recommendation is to incorporate these elements into the various mentioned stations, including CGS (City Gate Station), since these stations are positioned ahead of TBS and are more effective in capturing impurities. Enhancing the performance of the gas transmission lines can lead to overall improved efficiency. Additionally, the stations located within urban areas are likely to encounter fewer pollutants due to the cleaner gas supply from the prior stations, implementing these elements particularly advantageous in such urban settings.
4. Diverse varieties of nano filters can be manufactured and utilized under identical settings to evaluate and compare their industrial efficiency. Conducting experiments with multiple types of nano filters in the same operational conditions allows for a direct assessment of their performance and a thorough comparison of their effectiveness in industrial applications. This approach enables researchers and engineers to identify the most suitable nano filter design for specific industrial scenarios, optimizing pollutant capture and overall system efficiency.

## Data Availability

All data generated or analysed during this study are included in this published article.
