# Author Correction: The influence of nano filter elements on pressure drop and pollutant elimination efficiency in town border stations

**DOI:** 10.1038/s41598-023-49370-0

**Published:** 2023-12-19

**Authors:** Saeed Zeinali Heris, Hamed Ebadiyan, Seyed Borhan Mousavi, Shamin Hosseini Nami, Mousa Mohammadpourfard

**Affiliations:** 1 Xi’an University of Science and Technology, No. 58, Middle Section of Yanta Road, Xi’an, 710054 Shaanxi China; 2 Faculty of Chemical and Petroleum Engineering, University of Tabriz, Tabriz, Iran; 3 J. Mike Walker ‘66 Mechanical Engineering Department, Texas A&M University, College Station, TX 77843 USA; 4 School of Chemical, Biological and Materials Engineering, The University of Oklahoma, Norman, OK 73019 USA; 5 Department of Energy Systems Engineering, Izmir Institute of Technology, Izmir, Turkey

Correction to: *Scientific Reports* 10.1038/s41598-023-46129-5, published online 01 November 2023.

The original version of this Article contained an error in the order of the author names, which was incorrectly given as Hamed Ebadiyan, Saeed Zeinali Heris, Seyed Borhan Mousavi, Shamin Hosseini Nami & Mousa Mohammadpourfard.

Consequently, in the Author Contributions section,

“H.E. Investigation. S.Z.H. Supervision, Conceptualization, Methodology, Validation. S.B.M. Formal analysis, Writing original draft. S.H.N. Formal analysis, Writing original draft. M.M. Validation.”

now reads:

“S.Z.H. Supervision, Conceptualization, Methodology, Validation. H.E. Investigation. S.B.M. Formal analysis, Writing original draft. S.H.N. Formal analysis, Writing original draft. M.M. Validation.”

The original Article has been corrected.

